# Eye lens radiation exposure in paediatric interventional treatment of retinoblastoma

**DOI:** 10.1038/s41598-019-56623-4

**Published:** 2019-12-27

**Authors:** A. Obesso, L. Alejo, C. Huerga, F. Sánchez-Muñoz, E. Corredoira, A. Fernández-Prieto, R. Frutos, B. Marín, G. Garzón, J. Peralta, C. Ubeda, E. Guibelalde

**Affiliations:** 1 Medical Physics Department, La Paz University Hospital, Madrid, Spain; 2 Neuroradiology Department, La Paz University Hospital, Madrid, Spain; 3 Paediatric Ophthalmology Department, La Paz University Hospital, Madrid, Spain; 4 Medical Technology Department Health Sciences Faculty, Tarapaca University, Arica, Chile; 5 Radiology Department, Complutense University, Madrid, Spain

**Keywords:** Cancer prevention, Paediatric cancer

## Abstract

Retinoblastoma represents 3% of cancers in children under fifteen years of age. The standard paediatric treatment for saving the affected eye is supraselective intra-arterial chemotherapy performed in interventional rooms. In order to address the radiation toxicity due to the angiography, the aim of this study was to determine the typical dose value corresponding to the procedure, estimate the paediatric patients’ eye lens dose and study the relationship between dose indicators and dose to the lens. An automatic dose management software was installed in two interventional rooms to obtain the distribution of the dose indicators kerma-area product and reference-point air kerma, getting a typical value 16 Gy·cm^2^ and 130 mGy, respectively (n = 35). The eye lens dose estimates were obtained with photoluminescent dosimeters placed on the patient’s eyelids. In the left eye, the entrance surface air kerma was 44.23 ± 2.66 mGy, and 12.72 ± 0.89 mGy in the right eye (n = 10). There was a positive correlation between dose to the lens per procedure and dose indicators, with R^2^ > 0.65 for both eyes. Based on this information, the threshold for the onset of radiation-induce cataracts (500 mGy) will be exceeded if the treatment is performed for more than 8 sessions.

## Introduction

Retinoblastoma is a malignant tumour located in the retinal tissues and represents between 2.5% and 4% of paediatric cancers. Approximately 2 out of 3 cases are diagnosed before the age of 2 years, and 95% are diagnosed before the age of 5 years^1^. Currently, the treatment of choice in cases of advanced retinoblastoma grades higher than B (International Classification of Retinoblastoma^2^) is intra-arterial and fluoroscopy-guided chemotherapy performed in interventional rooms^3^, because recent studies have shown that intra-arterial chemotherapy is one of the most effective and least harmful treatments for retinoblastoma^3,4^. In fact, published data indicate eye survival rates of 85% for children under 2 years of age, 77% for children under 5 years and 71% for children under 7 years^4^. The alternative for these advanced cases are enucleation or external radiotherapy causing radiation-induced side-effects^3^. Given that this angiographic procedure is interventional and that children have an increased radiation sensitivity, the radiation doses need to be controlled and kept as low as possible without compromising the correct visualisation of the catheter, and preventing radio-induced cataracts^5,6^. An essential tool to optimise the radiation dose to the paediatric patients are the Diagnostic Reference Levels (DRLs), being the International Commission on Radiological Protection the responsible for proposing these DRLs for radiodiagnostic and interventional procedures^7–9^. When the median values of the dose indicators distributions provided by the X-ray equipment are significantly lower or higher than these levels, an investigation should be carried out in order to maintain the quality of the diagnostic information provided by the examination commensurate with the medical purpose while, at the same time, the patient exposures to radiation must be as low as reasonably achievable^7^. There is a limited number of articles related to estimating and calculating DRLs in paediatric interventional radiology^7,8^; however, there are publications on paediatric cardiac interventional procedures^10–12^. The paediatric treatment of retinoblastoma in the interventional neuroradiology room has yielded even fewer studies in the literature^13,14^. The application of the new European Directive 2013/59 EURATOM^15^ requires a regular review of the distributions of dose indicators in interventionism and optimisation of procedures without delay in cases in which DRLs are regularly exceeded. In addition, in *Article 61* paediatric interventional procedures are considered as “special practices” and therefore special attention shall be given to quality assurance programmes and the assessment of radiation dose. When only data of a single facility are available, the DRL is called *typical dose value* and is defined as the median of the dose indicators distribution. The first objective of our study was to obtain this typical dose value for treating paediatric retinoblastoma in terms of the most common radiation dose indicators in angiography: the air kerma-area product (P_KA_)^16^ and the reference-point air kerma (K_a,r_)^17^. The second study objective was to estimate the radiation dose to the lens for paediatric patients and propose optimisation measures to decrease this dose without compromising the clinical results of the intervention. The relationship between the dose to the lens and the dose indicators provided by the equipment was also analysed.

## Materials and Methods

### X-ray equipment, automatic dose management software and dosimeters

The interventional procedures were performed using two digital fluoroscopic X-ray systems: a Philips Allura FD20 monoplane system and a Philips Allura Clarity FD20/15 biplane system, both of which have a maximum output of 100 kW (1000 mA and 100 kV), a minimum exposure time of 1 ms, a voltage range of 40–125 kV, and a maximum current of 1250 mA at 80 kV. The standard protocol for both rooms is known as the “Cerebral” protocol, with 2 images per second, and the lowest level at 15 pulses per second. The frontal plane FD20 detector is the same in both systems and is rectangular and rotary, measuring 30 × 40 cm, with a 5-megapixel acquisition matrix with a 14-bit depth, and a pixel size of 0.154 × 0.154 mm. The FD 15 lateral plane detector of the Philips Allura Clarity is rectangular and measures 40 × 36 cm, with a pixel size of 0.184 × 0.184 mm and a 16-bit depth in acquisition. Both systems have a Spectra-Beam spectral filtering system to reduce radiation levels during fluoroscopy and an integrated ionization chamber that performs P_KA_ measurements during the procedure, and calculates the K_a,r_ from the generator’s load curves. These data are included in the dose structured report generated by the equipment, together with the parameters of each exposure (kV, mA, pulse size, field size, etc.).

The automatic dose management software DOLIR (Dose On-Line for Interventional Radiology), was developed by physicists at San Carlos Clinical Hospital in Madrid, Spain, and stores and statistically manages the information contained in the structured reports^18^. The application quickly obtains the local DRLs in terms of the dose indicators P_KA_ and K_a,r_, which are regularly verified and corrected using a semiconductor detector duly calibrated (Unfors Raysafe Xi Base Unit and R/F detector) and radiochromic film (Gafchromic XR-RV3), with tolerance results according to international protocols^19^.

The dosimetry equipment consisted of a series of Optically Stimulated Luminescence (OSL) dosimeters called nanoDots (Landauer Inc, IL, USA), an OSL reader (MicroStar, Landauer Inc.) and an external PC with custom software. NanoDots OSL dosimeters are made up of an active material (Al_2_O_3_:C) and measure 4 mm in diameter and 0.3 mm in thickness. When closed, the nanoDots are covered by a 10 × 10 × 2 mm light-proof plastic casing. This type of dosimeter has been employed in several studies to measure doses for patients and exposed workers in radiodiagnostic procedures^20–22^.

### Procedure description

Intra-arterial chemotherapy normally was applied in 3 interventions at 4-week intervals, although the number of sessions may vary depending on the response to treatment. Interventional neuroradiologists insert a 4 F catheter through the femoral artery (right or left, alternating for each session) to reach first the carotid artery, and then navigate a very soft flow-directed microcatheter (Magic 1.5 F, manufactured by Balt-Extrusion, Montmorency, France) to the ostium of the ophthalmic artery, through which finally inject the drugs. First images were taken by means of anterior-posterior (AP) projection. Once the internal carotid artery is reached, a lateral angiogram is performed with a radiation beam incident on the left side of the patient’s skull. This lateral angiogram is used as a road-mapping for the microcatheterism of the ophthalmic artery and to perform a selective lateral angiogram to confirm the existence of choroidal staining and flow to other branches. When it is not feasible to catheterise the ophthalmic artery, the microcatheter is navigated through the middle meningeal artery and its anastomosis with the ophthalmic artery as the chemotherapy is infused. The latter may require longer fluoroscopic and acquisition time. The drug commonly used is melphalan or this combined with topotecan and carboplatin^4^.

### Typical dose value calculation

The local DRLs must be established as the third quartile of the median values distribution of the patient dose indicators obtained from X-ray rooms within a few healthcare facilities in a local area. For this study, the only data considered were from the La Paz University Hospital, obtaining a typical value from the median of the dose indicators distributions provided by the automatic dose management software^7^. At La Paz University Hospital only 2% of the procedures performed in the interventional procedure rooms correspond to the treatment of this tumor, which represents 16 patients and their 35 corresponding interventions for treating retinoblastoma (1–4 sessions per patient). The DOLIR dose management software collected dosimetry and demographic data from March 2016 to February 2018.

### Eye lens dose estimate

In 10 of these 35 interventions for treating retinoblastoma, before starting the treatment, two OSLs were placed on the patient’s eyelids. This experimental set up was considered the best non-invasive method to estimate the dose to the lens —in fact the closest upper bound due to the small attenuation produced by the surrounding tissues. This approach is acceptable for radiation protection purposes, since allows to compare our estimates with the threshold dose value for the occurrence of the radio-induced cataracts. Also, the dosimeters are made of radiolucent material and therefore do not interfere with the tasks of the neuroradiologist. Therefore, we estimate the dose absorbed by the patient’s eye lens using the following expression for the entrance surface air kerma (K_a,e_)^16^:

1 $${{\rm{K}}}_{{\rm{a}},{\rm{e}}}={\rm{C}}\cdot {{\rm{N}}}_{{\rm{D}},{\rm{Q}}0}\cdot {\rm{S}}\cdot {{\rm{k}}}_{{\rm{Q}},{\rm{Q}}0}\cdot {{\rm{k}}}_{{\rm{a}}}$$

where C is the number of counts obtained by the reader’s photomultiplier; N_D,Q0_ is the calibration coefficient for the beam quality RQR6 (N_D,Q0_ = (1.41 ± 0.03)·10^–4^ mGy @ 80 kVp, average energy 44 keV and HVL of 3.01 mm Al)^23^; S is the sensitivity of the dosimeter as stated by the manufacturer, with a nominal uncertainty of 2%; and k_Q,Q0_ and k_a_ are the correction factors for energy and angularity, respectively (k_Q,Q0_ = 1.05 ± 0.04; k_a_ = 1.11 ± 0.07). The dosimeters were read 6 consecutive times, and the best estimate was considered to be the average of the last 5 measures. Therefore, the uncertainty of C was the quadratic sum of the type A uncertainties, due to the spread of the readings, and those of type B, due to the reader’s resolution and stability^21^.

### Correlation between the eye lens dose estimate and the dose indicators

In order to obtain a quick estimate of the paediatric patient’s eye lens dose, a correlation study between the left and right eye lens dose estimates obtained with nanoDots and the dose indicators P_KA_ and K_a,r_ was performed. As with the typical values, the P_KA_ and K_a,r_ values obtained in each procedure were verified and corrected *in situ* using the duly calibrated semiconductor detector and the radiochromic films. The corresponding correction factors varied by ± 6%, with an uncertainty of less than 3%. In the comparisons, the square of the Pearson coefficient was used.

### Ethical approval

This study was approved by the Ethics Committee of the La Paz University Hospital and performed in accordance with national regulations. All the corresponding informed consents was obtained from patient’s legal guardians.

## Results

### Typical dose values

**Figure 1:**
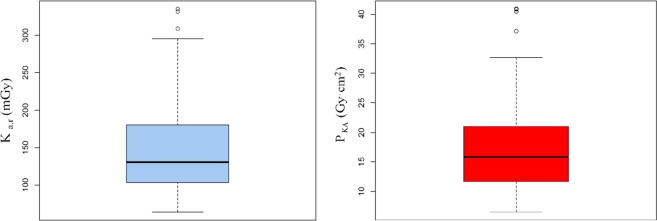
Dose indicators distribution in terms of K_a,r_ and P_KA_.

From March 2016 to February 2018, 16 paediatric patients were treated in the two fluoroscopic systems, with an age range of 0–5 years, and a mean age of 27 months, and 35 procedures were performed. From this data set, we obtained the median of the corresponding 35 DRL quantities, and thus the typical dose value, resulting a K_a,r_ of 130 mGy and P_KA_ of 16 Gy·cm^2^. In Fig. 1 the dose indicators values distributions are shown. For discussion purposes, the weighted average value of K_a,r_ was also obtained: 84.34 ± 0.55 mGy.

### Eye lens dose estimates

For the sample of 10 interventions used to estimate the eye lens dose, the age range of patients was between 0–4 years, and the mean age was 31 months. The patients had undergone an average of approximately 2 interventions. Table 1 lists the eye treated with intra-arterial chemotherapy during the intervention, the left and right eye lens doses obtained with OSL dosimeters, the corresponding values of the dose indicators and the patient’s weight. The weighted average estimated absorbed dose (K_a,e_) for the right and left eye lens per procedure was 12.72 ± 0.89 mGy and 44.23 ± 2.66 mGy, respectively, which was determined from the OSL readings (uncertainty expanded to k = 2).

**Table 1:** Estimates of the lens dose for both eyes performed with photoluminescent dosimeters, in terms of the entrance surface air kerma (K_a,e_).

K_a,e_ right (mGy)	K_a,e_ left (mGy)	Eye treated	Weight (kg)	P_KA_ (Gy·cm^2^)	K_a,r_ (mGy)
12.60 ± 0.42	40.96 ± 1.39	Left	11.3	14.40 ± 0.19	99.03 ± 0.74
14.27 ± 0.48	62.03 ± 2.10	Right	24.0	28.07 ± 0.36	181.15 ± 1.36
11.73 ± 0.40	52.16 ± 1.77	Right	13.4	11.36 ± 0.15	129.97 ± 0.98
9.70 ± 0.33	36.35 ± 1.23	Left	10.5	7.29 ± 0.10	94.70 ± 0.71
7.99 ± 0.27	37.54 ± 1.28	Left	18.3	9.34 ± 0.12	78.98 ± 0.59
12.33 ± 0.42	33.53 ± 1.14	Left	19.0	10.10 ± 0.13	78.12 ± 0.59
12.53 ± 0.47	30.52 ± 1.14	Left	8.5	10.00 ± 0.13	157.60 ± 1.19
42.61 ± 1.43	107.02 ± 3.62	Left	8.5	32.68 ± 0.42	295.49 ± 2.22
14.03 ± 0.47	39.03 ± 1.31	Left	13.0	15.97 ± 0.21	131.27 ± 0.99
8.62 ± 0.29	30.50 ± 1.02	Right	9.5	6.75 ± 0.11	75.23 ± 1.06

The eye treated in the procedure and the patient’s weight are shown, as well as the dose indicator values kerma-area product (P_KA_) and reference-point air kerma (K_a,r_).

### Correlation between the eye lens dose estimates and the dose indicators values

**Figure 2:**
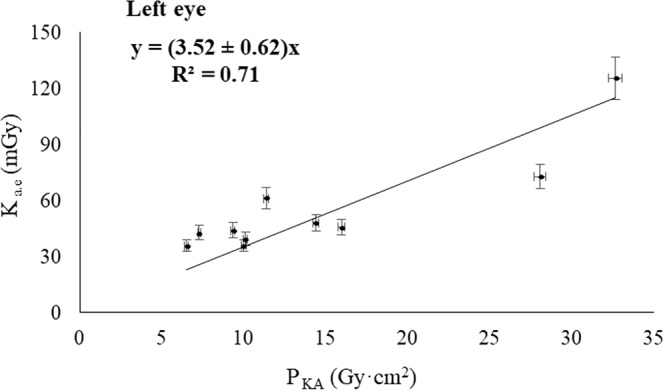
Correlation between K_a,e_ measured on the left eye and P_KA_.

**Figure 3:**
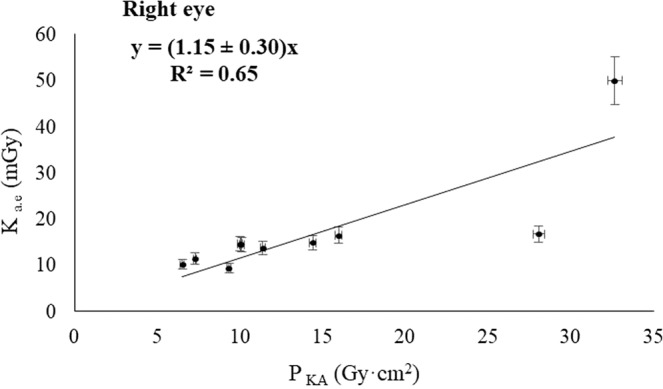
Correlation between K_a,e_ measured on the right eye and P_KA_.

**Figure 4:**
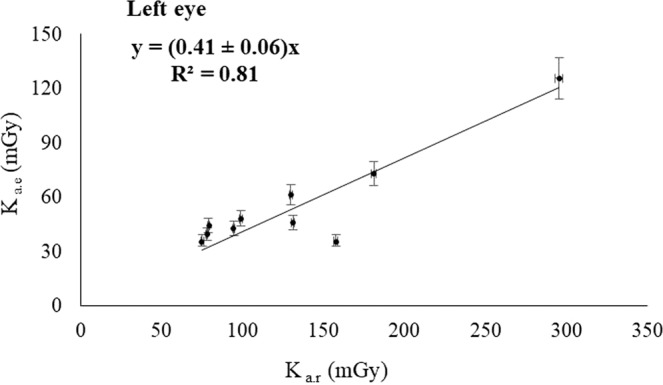
Correlation between K_a,e_ measured on the left eye and K_a,r_.

**Figure 5:**
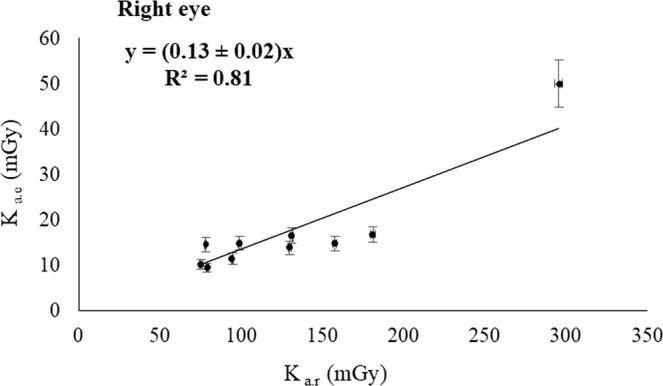
Correlation between K_a,e_ measured on the right eye and K_a,r_.

The Figs. 2, 3, 4 and 5 show the relationship between the corresponding values of the dose indicators and the eye lens dose estimates showed in Table 1. The Pearson correlation coefficient between K_a,e_ and P_KA_ was 0.71 for the left eye, and 0.65 for the right eye; and the Pearson coefficients between K_a,e_ and K_a,r_ were 0.81 for both eyes. The pending’ uncertainty is represented by the 95% confidence interval. Based on the number of interventions undergone by the patients recorded by the automatic dose management software, we can estimate the accumulated eye lens dose for each patient at the end of the treatment. The maximum cumulative eye lens dose per patient was found 250 ± 12 mGy and the dose for the corresponding healthy eye was 83 ± 5 mGy, which corresponds having undergone 4 interventions performed from December 2016 to April 2017. The mean values of the eye lens doses per procedure obtained by introducing the dose indicators in the lineal regressions with the highest R^2^ (Figs. 4 and 5) was 64 ± 2 mGy for the left eye and 21 ± 1 mGy for the right eye.

## Discussion

Although a single-centre experience result, the typical dose value we obtained for the treatment of retinoblastomas by supraselective intra-arterial chemotherapy represents a first approximation to the DRLs in paediatric interventional neuroradiology. These procedures are not common but can result in high doses of radiation in individual patients^8^. In our study we have shown that the patients’ eye lens radiation toxicity per procedure is not negligible. In fact, the estimated maximum cumulative dose in the eye lens for a patient who underwent 4 sessions corresponds to approximately 50% of the threshold dose for the onset of radiation-induced cataracts in the lens (500 mGy)^6^. The dose for the corresponding healthy eye corresponds to 17% of this threshold. The threshold for the onset of radiation-induced cataracts could therefore be exceeded if approximately 8 interventions were performed. Gobin *et al*.^4^ reported a range of 1–9 sessions of retinoblastoma treatment through intra-arterial chemotherapy for a sample of 229 patients aged between 1 month and 21 years (mean age 15 months). In addition, a recent occupational radiation exposure study shows excess risk for cataract associated with eye-lens absorbed dose lower than 100 mGy^24^. Currently we have no evidence of the appearance of cataracts in any of our paediatric patients, although the latency period has not yet been exceeded (2 to 3 years^25^).

The measurements performed with OSL dosimeters indicate that the dose absorbed by the left eye was always higher than that absorbed by the right eye, regardless of which eye was treated (Table 1). This result could be because the X-ray tube was always located in the same position when taking the lateral images. The neuroradiologist is usually to the right of the patient, and the image plane is rotated in such a way that the scattered radiation received by the physician is as little as possible. If the plane were turned in the opposite direction when the right eye is to be treated, this might optimise the dose received by the left eye. In our case, this approach could be recommended if more than 8 treatment sessions are expected (i.e. when it is possible to exceed the dose limit for the occurrence of the radio-induced cataracts). However, for radiation protection purposes, this new beam configuration would imply to change the position of the neuroradiologist to the left of the patient, or the use of additional radiation protection tools. Therefore, this optimisation strategy could have clinical implications, and it will be the subject of further studies. Another possible optimisation measure is the placement of lead protectors on the patient’s eyes. However, the difference in dose received by the two eyes (3–5 times greater in the left eye than in the right) is probably due to the fact that maximum radiation is received during lateral projections. Also, for AP projections the automatic exposure control system would significantly increase the dose by introducing a radiopaque element in the central area of the field, rendering the protection tool counterproductive. Additionally, usual strategies of optimization should be applicable, as the use of specific low dose paediatric protocols, further collimation to reduce the field of view, placing the image detector as close to the patient as possible, removing the anti-diffusing grill and performing ocular angiography by subtracted fluoroscopy instead of angiography by digital subtraction^5^.

A positive correlation was found between the estimated doses absorbed by the patient’s lens and the dose indicators provided by the automatic dose management software, with the square of the Pearson coefficients ranging 0.65–0.81. Increasing the statistical confidence of the results would require more estimates with dosimeters placed in the vicinity of the patient’s eyes. Due to the positive correlation observed between the magnitudes, we could estimate the patient’s eye lens dose without placing dosimeters on the eyes during the procedures. Therefore, the linear regressions could be useful in monitoring the progression of the patient’s vision. Based on these correlations, we can tell whether a patient who has undergone several interventions has a cumulative eye lens dose close to the threshold for the onset of radiation-induced cataracts. These results can be of application in other interventional suites provided that the dose indicators P_KA_ and K_a,r_ have been corrected using multimeter and radiochromic films duly calibrated, and the dose to the lens have been estimated using dosimeters duly calibrated likewise. The geometry of the interventionist arc must also be considered so the resulting regression model was properly applied.

As previously mentioned, the literature regarding this type of treatment is limited. However, there are studies with which we can compare results (Table 2). Cooke *et al*.^26^ and Gobin *et al*.^27^ obtain eye lens dose values 1–2 orders of magnitude lower than those of this work, possibly due to they had already performed a dose optimisation process. However, our results are similar to that of Thampi *et al*.^14^, and considerably lower than Vijayakrishnan *et al*.^25^. Therefore, high variability is observed in the literature in terms of patients’ eye lens dose, and this issue is possibly related to the optimization of the fluoroscopic and acquisition protocols, as well as the experience and the methods used by the physicians involved in the retinoblastoma treatment. In terms of the dose indicator or DRL quantities, we could compare our weighted average reference-point air kerma with the peak skin dose for the most common interventional radiology procedures (such as embolizations or angiographies) presented by Orbach *et al*.^28^, Realson *et al*.^29^ and Thierry-Chef *et al*.^30^. The corresponding skin dose values were one order of magnitude greater than the reference-point air kerma obtained in this study (the K_a,r_ can be considered an upper bound of the peak skin dose). This may be due to the high complexity of the interventional procedures considered. In terms of the P_KA_, Boddu *et al*.^31^ presents a result for mean value for the retinoblastoma treatment with similar order of magnitude as our weighted average. Finally, the European Guidelines on Diagnostic Reference Levels for Pediatric Imaging^8^ presents a < P_KA_ > _3/4_ for head embolization an order of magnitude greater than our result.

**Table 2:** Paediatric patients’ eye lens dose per procedure, number of studies and average age reported by different authors.

	Average age (months)	N° studies	K_a,e_ (mGy)
Cooke *et al*.^26^	24	4	0.18 ± 0.10
Thampi *et al*.^14^	15	16	20.2 ± 11.9
Gobin *et al*.^27^	15.5	16	0.99
Vijayakrishnan *et al*.^25^	29	11	166.8
This study	27	35	Left: 64 ± 2
			Right: 21 ± 1

## Conclusions

A typical dose value for the retinoblastoma treatment by intra-arterial supraselective chemotherapy in paediatric patients has been determined, obtaining P_KA_ = 16 Gy·cm^2^ and K_a,r = _130 mGy (n = 35). In 10 interventions, the dose absorbed by the patients’ eye lens was estimated with OSL dosimeters, resulting in mean values of 44.23 ± 2.66 mGy for the left eye and 12.72 ± 0.89 mGy for the right eye. A good approximation of the estimated dose absorbed by the lens was obtained using dose indicators provided by the automatic dose management software, with correlation coefficients close to 1. Based on this information, we can deduce that the threshold for the onset of radiation-induced cataracts will be exceeded if the retinoblastoma treatment guided by intra-arterial chemotherapy is performed for more than 8 sessions.
